# Increased prevalence of the founder *BRCA1* c.5309G>T and recurrent *BRCA2* c.1310_1313delAAGA mutations in breast cancer families from Northerstern region of Morocco: evidence of geographical specificity and high relevance for genetic counseling

**DOI:** 10.1186/s12885-023-10822-5

**Published:** 2023-04-13

**Authors:** Rahma Melki, Marouane Melloul, Souria Aissaoui, Tijani EL Harroudi, Noureddine Boukhatem

**Affiliations:** 1 Genetics and Immune-Cell Therapy Unit, LBBES Laboratory, Faculty of Sciences, University Mohammed Premier, Oujda, Morocco; 2 Microbiology and Molecular Biology Unit, PMBBE Center, Faculty of Sciences, Mohammed V University, Rabat, Morocco; 3 Genetics Service of Genesuport, Geneva, Switzerland; 4 Surgical Oncology, Faculty of Medicine and Pharmacy, University Mohammed Premier , Oujda, Morocco

**Keywords:** Breast cancer, *BRCA1*, *BRCA2*, Mutation, Moroccan population

## Abstract

**Background:**

Inherited mutations in the breast cancer susceptibility genes *BRCA1* and *BRCA2* (*BRCA1/2*) confer high risks of breast and ovarian cancer. Because the contribution of *BRCA1/2* germline mutations to BC in the Northeastern population of Morocco remains largely unknown, we conducted this first study to evaluate the prevalence and the phenotypic spectrum of two *BRCA1/2* pathogenic mutations (the founder *BRCA1* c.5309G>T and *BRCA2* c.1310_1313delAAGA). This choice was also argued by the presence of an apparent specific geographical connection of these mutations and the Northeastern region of Morocco.

**Methods:**

Screening for the germline mutations c.5309G>T and *BRCA2* c.1310_1313delAAGA was performed by sequencing on a total of 184 breast cancer (BC) patients originated from the Northeastern region of Morocco.

The likelihood of identifying a BRCA mutation is calculated using the Eisinger scoring model. The clinical and pathologic features were compared between the BRCA-positive and BRCA-negative groups of patients. Difference in survival outcomes was compared between mutation carriers and non-carriers.

**Results:**

*BRCA1* c.5309G>T and *BRCA2* c.1310_1313delAAGA are responsible for a significant proportion of all BC cases (12.5%) and at least 20% of familial BC. The screening of *BRCA1/2* genes by NGS sequencing confirmed that there are no additional mutations detected among positive patients.

The clinicopathological features in positive patients were in accordance with typical characteristics of BRCA pathogenic mutations. The mean features in the carriers were the early onset of the disease, familial history, triple negative status (for *BRCA1* c.5309G>T) and worse prognosis in terms of overall surviving.

Our study indicates that the Eisinger scoring model could be recommended to identify patients for referral to *BRCA1/2* oncogenetic counseling.

**Conclusion:**

Our findings suggest that *BRCA1* c.5309G>T and *BRCA2* c.1310_1313delAAGA mutations may have a strong founder and/or recurrent effect on breast cancer among the Northeastern Moroccan population. There contribution to breast cancer incidence is certainly substantial in this subgroup. Therefore, we believe that *BRCA1* c.5309G>T and *BRCA2* c.1310_1313delAAGA mutations have to be included in the array of tests aimed at revealing cancer syndrome carriers among subjects of Moroccan origin.

## Background

The vast majority of breast cancer (BC) cases are considered sporadic-appearing tumors for which environmental and life-style factors are the most important determinants of the risk, while 5% to10% of all cases are thought to develop because of a genetic predisposition [1].

Hereditary Breast and Ovarian Cancer (HBOC) is a genetic predisposing syndrome characterized by a young age of onset, a type of tumor as well as family history. HBOC like most of the other genetic predisposing syndromes to cancer is caused by germline mutations of oncogenes and tumor suppressor genes. Deleterious germline mutations of at last 15 genes (*BRCA1/2*, ATM, BARD1, CDH1, CDKN2A, CHEK2, MLH1, MSH2, MSH6, NF1, PALB2, PTEN, RAD51D, TP53, BRIP1) are associated with an increased risk (> 2.0 times) of breast cancer. Deleterious germline mutations of eleven genes are associated with an increased (> 2.0-fold) risk of ovarian cancer (ATM, *BRCA1* / 2, BRIP1, MSH2, MSH6, NBN, PMS2, RAD51C, RAD51D, and TP53) [2].

Breast Cancer 1 gene (BRCA1) and Breast Cancer 2 gene (BRCA2) are included in the category of high penetrance genes. A plethora of germline pathogenic mutations of *BRCA1/2* genes (over 3300) which are inherited in an autosomal dominant form are responsible for the major HBOC cases ( http://www.hgmd.cf.ac.uk/ac/index.php). These variants are closely related to high lifetime risk of developing HBOC; the reported cumulative lifetime risk of breast cancer is approximately 72% and 69%, up to the age of 80 years, for *BRCA1* and *BRCA2* respectively [3].

Both *BRCA1* and *BRCA2* are tumor suppressor genes, and are instrumental in a range of cellular regulating pathways, including regulating DNA double-strand breaks repair in the process of homologous recombination, genomic integrity, transcriptional regulation, apoptosis, chromosomal segregation and chromatin remodeling [4, 5]. There is also evidence that *BRCA1* is an important link in the signal chain that starts with recognition of DNA damage (sensed by ATM) and leads to cell cycle arrest at the G2/M checkpoint [6].

*BRCA1/2* mutations exhibit important differences in prevalence and spectrum across various racial/ethnic groups and geographical regions. In some ethnic communities or specific populations, mutations of *BRCA1/2* genes are more frequent due to founder effects. This is particularly remarkable in Ashkenazi Jews population, Polish, Norwegian, Icelandic people and in several other area where isolated populations exists [1, 7, 8].

With the emergence of genetic testing, *BRCA1/2* profiling was strongly recommended for women with a family history or early age onset of BC [9]. The assessment of *BRCA1/2* mutation carriers in familial breast cancer has been proved to be valuable not only in the perspective of prevention and early detection of related cancers but also it has implications in implementation of personalized medicine and chemoprevention of recurrence. It has been suggested that breast cancer patients with *BRCA1/2* mutations may benefit from precision treatments, such as platinum-based chemotherapy and poly ADP-ribose polymerase inhibitors [10]. On another side, the identification of the most prevalent or founder mutations in an ethnic population will facilitate earlier and rapid and especially cheaper molecular diagnosis of BC. Therefore, these have made it imperative for the recurrent and founder mutations of the *BRCA1/2* genes within low income countries to be identified and included in breast cancer screening and diagnosis [11].

Although the highest rates of breast cancer incidence are observed in developed countries, the incidence of this disease has clearly risen in Arab countries including Morocco, a country of North-western Africa. BC is still a major cause of death by cancer among Moroccan women and accounted for 35.8% of all registered cancers. The latest statistics available reported an increasing incidence rate from 39.0 to 49.5 per 100.000 women in this population between 2008 and 2012 [12]. It remained the highest incidence among the countries of North-western Africa (Algeria and Tunisia) [13]. Data reported in Moroccan population, showed a higher proportion of BC among young women aged between 45 to 49 years, and a frequent clinical observation of family history. All this suggests a strong influence of high-penetrance genetic factors in BC etiology [14, 15].

To date, published studies on the contribution of *BRCA1/2* mutations to BC in the Moroccan population are still limited. Only a partially characterized *BRCA1* mutation landscape in BC Moroccans is available and includes the following deleterious mutations: c.68-69delAG, c.116G > A, c.181 T > G, c.798-799delTT, c.1016dupA, c.2126insA, c.2805delA, c.3279delC, c.3453delT, c.4942A > T, c.5062-5064delGTT and c.5095C > T [16–19]. The main pathogenic mutations detected in *BRCA2* gene were c.289G > T, c.517-1G > A, c.1310_1313delAAGA, c.3381delT, c.3847_3848delGT, c.5073dupA, c.5116_5119delAATA, c.5576-5579delTTAA, c.6428C > A, c.7110delA and c.7234_7235insG [16, 17, 19–21]. For all of these mutations, there is no evidence provided on their founding effect in the Moroccan population. Furthermore, all of patients recruited in these previous studies have been diagnosed in few cancer institutions based primarily in the west of Morocco (mainly in the two large cities of Casablanca and Rabat), and therefore are not fully representative of the whole population. Interestingly and independently of the studies carried out in Morocco, another Spanish study, conducted by Quiles et al. [22], reported a new *BRCA1* deleterious mutation with founder effect (c.5309G>T, G1770V) in five families of Moroccan origin but settled in Spain and Norway. Interestingly, all of the five independent families were originated from the same area of Morocco, mainly from the regions of Oujda and Nador located in Northeastern Morocco. Most importantly, this founder mutation has not been described in previous published Moroccan studies. Otherwise, another mutation, c.1310_1313delAAGA located on *BRCA2* gene, was first described in Morocco in 2016 and 2017 [23, 24]. These studies which focused on a total of 122 patients originating from different regions of Morocco showed that 9 out of 14 positive cases (64.3%) shared the same geographic origin in the Northeast of Morocco essentially from the region of Oujda Angad; the other five patients (35.7%) were from neighboring central regions.

In the light of these observations, it seems essential to us to undertake the present study on a cohort of pathologically confirmed female breast cancer patients who originated from the Moroccan Northeastern region. Our intention was not only to ascertain the specificity and prevalence of the two *BRCA1/2* mutations (BRCA1, c.5309G>T, and BRCA2, c.1310_1313delAAGA) in the Moroccan Northeastern region, but also to evaluate their role in tumor phenotypic spectrum and disease prognosis, and to establish an adapted and rapid procedure for *BRCA1/2* mutations screening among the population of this region for better clinical management of BRCA mutation carriers.

## Methods

### Study participants

We established a cohort including 184 pathologically confirmed female breast cancer patients who originated from the Moroccan Northeastern region. Four BC male patients were also included. All patients were referred from the Hassan II Regional Oncology Center of Oujda. The study protocol was reviewed and approved by the ethics committee for Biomedical Research of the Faculty of Medicine and Pharmacy of Casablanca under the number 06/18. Written informed consent for research participation was obtained from all subjects prior to peripheral blood collection.

Clinical and pathological data were abstracted from patient’s medical files and pathology reports. The recorded information included age at diagnosis, family history of breast cancer, laterality, tumor histology type, Scarff-Bloom-Richardson (SBR) grade, tumor size, lymph node involvement, metastases, survival as well as hormone receptor status including: estrogen receptor (ER), progesterone receptor (PR), and human epidermal growth factor receptor 2 (Her-2).

### Eisinger score carrier risk prediction

Germline mutations in *BRCA1/2* genes have important implications for treatment of patients diagnosed with breast or ovarian cancers as well as unaffected carriers of these mutations. Various statistical models have been established to predict the likelihood of identifying a deleterious BRCA mutation based on an individual's personal and family history [25].

According to the criteria published by the national expertise INSERM-FNCLCC in 2004 based on personal and familial breast and ovarian cancer history, we used the Eisinger scoring system that has been used in Europe to calculate the likelihood of carrying a *BRCA1/2* pathogenic variants for each patient [26]. The Eisinger score (seven factors, score from 0 to 5) is a simple family tree analysis risk assessment tool to validate the indication for an oncogenetic consultation and to consider a search for mutations. It also helps to gradate the risk of genetic predisposition to breast cancer in the absence of identified familial mutation (score = 5 or more: excellent indication; score = 3 or 4: possible indication; score = 1 or 2: no indication). Based on the calculation of Eisinger score for each patient in our cohort, 46.15% of the patients have criteria and personal and/or familial history suggesting an increased risk of breast cancer due to genetic predisposition (familial group; Eisinger score > 3). The other 53.85% of the patients have no such risk criteria and the cancers may be classified as sporadic (sporadic group; Eisinger score ≤ 3).

### Molecular Analysis of BRCA1/2 genes

Peripheral blood samples from the patients were collected in EDTA coated tubes. Genomic DNA extraction was done using a standard salting-out method [27]. DNA concentration and quality were evaluated by NanoVue Plus™ spectrophotometer (biochrom, Harvard Bioscience Inc. Massachusetts, USA), and stored at − 20 °C until analysis.

The target screening of the c.5309G>T founder mutation was performed by PCR-based Sanger sequencingof *BRCA1* exon 21. Amplification and sequencing of a 320 bp fragment were carried out with the following primers: forwardprimer 5’-cttgtccctgggaagtagca-3’and newly designed reverse primer 5’-gatgggggttcctcagattg-3’ (designed through Primer 3 software) [28]. To screen for c.1310_1313delAAGA mutation at exon 10 of BRCA2, a PCR product of 552 bp was amplified and sequenced with the following primers: forward primer 5’-tggaaccaaatgatactgatcc-3’and reverse primer 5’- cctctgaaagtggactggaaa-3’.

The PCR was performed in a volume of 25 μL. Cycling conditions were 94 °C for 30 s, 60 °C for 30 s, and 72 °C for 30 s for 30 cycles in the case of *BRCA1* exon 21. Thirty five cycles of 94 °C for 30 s, 58 °C for 30 s, and 72 °C for 30 s were cycling conditions used for *BRCA2* exon 10. Purifcation of the PCR products was done by ExoSAP-IT purifcation kit (Thermo Fisher Scientifc, Waltham, MA) according to the manufacture’s protocol. Purified products were sequenced bidirectionally using the Big Dye Terminator Cycle Sequencing kits ((Life technologies, Inc. Foster City, CA) and the same primers used for PCR. The sequence data were collected from an automated ABI Prism 3130XL capillary electrophoresis system (Life technologies, Inc. Foster City, CA). Alignment to the *BRCA1* and *BRCA2* reference genomic sequence (GenBank entries: NM_007294 and NM_000059 respectively) was done with SeqScanner v2 software (Applied Biosystems).

### Next generation sequencing

Germline mutation profiling using NGS was performed in blood samples of 54 patients belonging to the present cohort and including all patients carrying the mutations *BRCA1* c.5309G>T and *BRCA2* c.1310_1313 DelAAGA.

*BRCA1* and *BRCA2* genes were screened by sequencing using Ion Proton next generation sequencing platform (Thermo Fisher Scientific). The NGS library was constructed using the Oncomine BRCA Research Assay, Chef-Ready Library Preparation (IonTorrent, Thermo Fisher Scientific, USA) according to the manufacturer’s instructions. Reads were aligned to the human genome reference sequence 19 (hg19) and variant identification were performed with the Torrent Suite v.5.12 software. The generated BAM files of each sample were imported to the Ion Reporter Software v5.18 and run using the Oncomine BRCA Research Germline workflow for variants annotations. Exonic sequence analysis was performed with average depth coverage of at least 200X.

### Statistical analysis

The clinical and pathologic features were compared between the BRCA-positive and BRCA-negative groups of patients. Descriptive of clinical data were expressed in percentage or mean ± SD. The statistical significance of associations was evaluated for categorical variables based on Pearson’s chi-squared test or Fisher’s exact test. The Mann–Whitney test was used to compare the mean ages of the different groups. The *p* values were based on two sided tests and conducted at a 5% significance level. Statistical analyses were performed using SPSS software (version 21.0; IBM Corp. Armonk, NY, USA).

### Overall survival

Overall survival (OS) was determined as the length of time from the date of diagnosis until either the date of death or the date of last follow-up. We estimated the survival function with the Kaplan–Meier estimator. Difference in survival outcomes between mutation carriers and non-carriers was compared with the Log-Rank test.

## Results

### Patient’s characteristics

The mean age of patients at the time of diagnosis was 42.3 ± 0.72 years (range from 25 to 72 years). Personal and family history informations of BC were used in the Eisinger score calculation to distinguish two groups of patients. The first group (47%) included female patients with personal and/or familial history suggesting an increased risk of breast cancer due to genetic predisposition (score > 3). We called this group “Familial”. The second group (53%) included patients who have no personal and/or familial cancer history that may suggest a genetic predisposition to breast cancer (score ≤ 3). We named this group “sporadic”. The Table 1 showed the clinical characteristics of BC in these two groups of patients. Clinicopathological data were not available for two positive female BC. The mean age at diagnosis was 38.6 ± 0.95 years and 45.6 ± 0.95 years for familial and sporadic groups respectively. There are more patients under 39 years of age in the familial group compared to the sporadic group (55.3% vs. 28.9%; *p* = 0.0003). Patients with bilateral breast cancer (9.4%) were found only in the familial group (8/85). There is no case of bilateral breast cancer in the sporadic group (*p* = 0.002). In addition, early stage tumor (T2) and histological grade III were slightly more frequent in patients with familial history compared to sporadic group (*p* = 0.03 and 0.04 respectively). Contrariwise, the proportion of patients developing metastasis and lymph node involvement was slightly higher in the latter group (0.026 and 0.001 respectively). Finally, there was no significant difference regarding histological type of the tumor and hormone receptor expression between the two groups of patients.

**Table 1:** Comparison of clinicopathological features between familial and sporadic patients groups

**Patients Groups**	**Familial**	**Sporadic**	
**Clinical features**^**b**^	**N (%)**	**N (%)**	***P***^a^
**Age at diagnosis**			
Mean (years ± SD)	38,58 ± 0.95	45.62 ± 0.95	** < *10***^***–6***^
(Min–Max)	(25–71)	(30–72)	
≤ 39	47 (55.3)	28 (28.9)	
> 39	38 (44.7)	69 (71.1)	***0.0003***
***BRCA1* c.5309G>T**			
Positive	9 (10.7)	1 (1.1)	***0.007***
Negative	75 (89.3)	91 (98.9)	
***BRCA2* c.1310_1313 DelAAGA**			
Positive (total: 12)***	7 (11.9)	3 (3.9)	*0.1*
Negative	52 (88.1)	74 (96.1)	
**Both mutations**			
Positive (total: 22)***	16 (19)	4 (4.2)	***0.002***
Negative	68 (81)	91 (95.8)	
**Laterality**			
Bilateral	8 (9.4)	0 (0)	
Unilateral	77 (90.6)	97 (100)	***0.002***
**Histological type**			
Ductal invasive	71 (83.5)	83 (85.6)	
Lobular invasive	8 (9.4)	9 (9.3)	*0.86*
others	6 (7.1)	5 (5.2)	
**Histological grade**			
I + II	59 (69.4)	79 (82.3)	
III	26 (30.6)	17 (17.7)	***0.04***
**RE**			
Positive	63 (75)	67 (69.8)	
Negative	21 (25)	29 (30.2)	*0.44*
**RP**			
Positive	59 (70.2)	59 (61.5)	
Negative	25 (29.8)	37 (38.5)	*0.22*
**Her-2**			
Positive	17 (21.5)	18 (18.8)	
Negative	62 (78.5)	78 (81.2)	*0.65*
**Sub-Type**			
Luminal	44 (55.7)	63 (65.6)	
Her-2 +	16 (20.3)	18 (18.8)	*0.31*
Triple Negative	19 (24.0)	15 (15.6)	
**Tumor size**			
T1	20 (24.1)	17 (18.1)	
T2	53 (63.9)	51 (54.3)	***0.03***
T3-T4	10 (12)	26 (27.7)	
**Node**			
N +	54 (64.3)	76 (79.2)	
N0	30 (35.7)	20 (20.8)	***0.026***
**Metastase**			
M0	80 (95.2)	74 (77.1)	
M +	4 (4.8)	22 (22.9)	***0.001***

*N (%)* number and percentage of individuals (except for mean ± SD age), *SD* Standard deviation, *Her-2* Human epidermal growth factor receptor-2, *PR* Progesterone receptor, *ER* estrogen receptor, *TN* Triple negative

Unknown data were excluded from statistical analysis

^a^bold values are statistically significant (*P* < 0.05)

^b^ Clinicopathological data are missing for two patients; therefore, these are not included in the calculations

The 4 males BC are not included in this analysis

### BRCA1 c.5309G>T and BRCA2 c.1310_1313 DelAAGA mutations

As indicated in Table 1, the overall prevalence of pathogenic *BRCA1* c.5309G>T and *BRCA2* c.1310_1313 DelAAGA mutations among east-Moroccan BC female patients was 22 (12.15%). The large and significant proportion was observed in familial group compared to sporadic one (16, (19%) and 4 (4.2%) respectively; *p* = 0.002). All positive cases were heterozygous for these mutations. The screening of *BRCA1/2* genes by NGS sequencing confirmed that there are no additional mutations detected among the mutation carrier patients.

### *BRCA1 *c.5309G>T mutation

The heterozygous *BRCA1* c.5309G>T mutation was found in 10 patients (5.43%). the frequency of the mutation was significantly higher in familial BC group (9, (10.1%)) than in sporadic group (1, (1.1%) (*p* = 0.007).

### *BRCA2* c.1310_1313 DelAAGA mutation

In total, we identified 12 females (7.35%) carrying *BRCA2* c.1310_1313 DelAAGA mutation. The highest prevalence of the mutation was observed in the in familial BC group (7, (10.1%)) than in sporadic group (3, (1.1%)). Data are missing for two patients; therefore these are not included in the calculations. Interestingly, the mutation was also encountered in two patients among four BC males.

### Clinical features of *BRCA1* c.5309G>T associated BC

Detailed clinicopathological features of 12 positive females BC and two males BC are listed in Table 2. The comparisons between female carriers and non carriers are summarized in Table 3. The average of age at diagnosis in patients with c.5309G>T mutation was 41 years (SD = 6.02, range: 33–50). Forty percent of carriers showed an age of onset below 39 years comparatively to the non carriers. There are also 50% who were triple negative. Most of patients (80%) shared similar histopathological features including unilateral BC, ductal invasive histological type and tumor grades I-II. Moreover, Table 3 showed that 55.6% of carriers had intermediate tumor lesion (T2) and positive lymph nodes. But only 20% developed metastasis. The distribution of these features in the two groups of patients was equivalent.

**Table 2:** Clinical and pathological characteristics of *BRCA1* c.5309G>T and *BRCA2* c.1310_1313 DelAAGA mutation carriers in Moroccan population

Patient	Age at diagnosis	Laterality	Tumor Type	Histological grade	ER PR Her-2 status	TNM	Sub-type	Family history	Predicted BRCA mutation Risk^a^
***BRCA1* c.5309G>T mutation**									
1	36	unilateral	IDC	II	+ + -	pT2 N0 M0	luminal	Twin sister (33 y); 2 cousins (34 y)	7
2	34	bilateral	IDC	III	- - -	M +	TN	Sister (OC); Father (BC); 3 sisters; 2 cousins	16
3	42	unilateral	IDC	I	+ + -	pT1 N + M0	luminal	2 sisters died	6
4	50	bilateral	IDC/IMC	III	+ + -	pT2 N + M0	luminal	Aunt; cousin (OC)	6
5	46	unilateral	ILC	II	- - -	pT2 N0 M0	TN	Sister (30 y); cousin (30 y); cousin (28 y)	8
6	33	unilateral	IDC	II	- - -	pT2 N + M0	TN	Twin sister (36 y); 2 cousins (34 y)	7
7	42	unilateral	IDC	II	- - -	pT3N + M0	TN	Sister (29 y); sister (40 y)	8
8	49	unilateral	IDC	II	- - -	pT2 N0 M0	TN	Sister; 3 nieces	10
9	37	unilateral	IDC	II	+ - -	pT1 N0 M0	luminal	Cousin (34 y)	4
10	41	unilateral	IDC	II	+ + -	pT3 N + M1	luminal	No family history	2
***BRCA2* c.1310_1313 DelAAGA mutation**									
11	32	unilateral	IDC/ILC	II	+ + -		luminal	Aunt died	3
12	30	unilateral	IDC	III	+ - -	pT2N + M0	luminal	Sister; 3 nieces	9
13	37	unilateral	ILC	II	+ + -	pT2N + M0	NA	Sister; M. cousin	4
14	45	unilateral	IDC	II	- - -	pT2N + M0	TN	Sister (31 y); M. and P. cousin	6
15	30	unilateral	IDC	II	+ + -	pT1N + M0	luminal	Sister (43 y); aunt (50 y)	5
16	28	unilateral	IDC	II	+ + -	pT1N + M0	luminal	Aunt (OC)	7
17	NA	NA	NA	NA	NA	NA	NA	NA	NA
18	47	unilateral	IDC	III	+ + -	pT2N + M0	luminal	Sisters; aunt; cousin (43 y)	6
19	NA	NA	NA	NA	NA	NA	NA	NA	NA
20	36	unilateral	IDC	I	- - -	pT2N + M0	TN	Aunt (young); cousin	5
21	54	unilateral	IDC	III	+ + -	pT2N + M0	luminal	NA	NA
22	46	unilateral	IDC	III	+ - -	pT1N0M0	luminal	NA	NA
1 (male)	65	unilateral	IDC	II	+ + -	pT2N + M0	luminal	Sister; 3 nieces died (BC)	8
2 (male)	71	unilateral	IDC	II	+ + -	pT2N + M0	luminal	3 nieces	7

*IDC* Invasive ductal carcinoma, *ILC* Invasive lobular carcinoma, *IMC* Invasive medular carcinoma, *TN* Triple Negative, *TNM* Tumor size, Node, Metastase, *BC* Breast cancer, *OC* Ovarian cancer, *M* Maternal, *P* Paternal

^a^Predicted BRCA mutation risk was calculated by Eisenger score [26]

*NA* Not available

**Table 3:** Comparaison of clinicopathological features by germline *BRCA1* c.5309G > T *and BRCA2* c.1310_1313 DelAAGA mutations in female BC of northeastern Morocco

	***BRCA1* c.5309G>T mutation**			***BRCA2* c.1310_1313 DelAAGA mutation**		
	**Neg (*n* = 174)**	**Pos (*n* = 10)**		**Neg (*n* = 132)**	**Pos (*n* = 12)**	
**Characteristics**	**n (%)**	**n (%)**	***p***^**a**^	**n (%)**	**n (%)**	***p***^**a**^
**Age at diagnosis**						
Mean (years ± SD)	42.2 ± 9.62	41 ± 6.02	*0.799*	43.96 ± 9.89	38.5 ± 8.92	*0.09*
≤ 39	70 (41.9)	4 (40)		47 (37)	6 (60)	
> 39	97 (58.1)	6 (60)	*1*	80 (63)	4 (40)	*0.185*
unknown	7	0		5	2	
**Family history of BC**^**b**^						
Sporadic	91 (54.8)	1 (10)		74 (58.7)	3 (30)	
Familial	75 (45.2)	9 (90)	***0.007***	52 (41.3)	7 (70)	*0.1*
**Laterality**						
Bilateral	6 (3.6)	2 (20)		8 (6.3)	0 (0)	
Unilateral	161 (96.4)	8 (80)	*0.067*	119 (93.7)	10 (100)	*1*
**Histological type**						
Ductal invasive	140 (83.8)	8 (80)		108 (85)	8 (80)	
Lobular invasive	16 (9.6)	1 (10)	*1*	11 (8.7)	1 (10)	*1*
others	11 (6.6)	1 (10)	*0.51*	8 (6.3)	1 (10)	*0.5*
**Histological grade**						
I + II	125 (75.3)	8 (80)		97 (77)	6 (60)	
III	41 (24.7)	2 (20)	*1*	29 (23)	4 (40)	*0.256*
**RE**						
Positive	120 (72.7)	6 (60)		89 (71.2)	8 (80)	
Negative	45 (27.3)	4 (40)	*0.47*	36 (28.8)	2 (20)	*0.725*
**RP**						
Positive	109 (66.1)	6 (60)		83 (66.4)	6 (60)	
Negative	56 (33.9)	4 (40)	*0.74*	42 (33.6)	4 (40)	*0.735*
**Her-2**						
Positive	34 (21.2)	0 (0)		24 (19.8)	0 (0)	
Negative	126 (78.8)	10 (100)	*0.21*	97 (80.2)	9 (100)	*0.209*
**Sub-Type**						
Luminal	97 (60.6)	5 (50)	*Ref*	75 (62)	7 (77.8)	*Ref*
Her-2 +	33 (20.6)	0 (0)	*0.33*	23 (19)	0 (0)	*0.343*
Triple Negative	30 (18.8)	5 (50)	*0.12*	23 (19)	2 (22.2)	*1*
**Tumor size**						
T1	33 (20.2)	2 (22.2)	*1*	28 (22.8)	3 (30)	*0.722*
T2	95 (58.3)	5 (55.6)	*Ref*	68 (55.3)	6 (60)	*Ref*
T3-T4	35 (21.5)	2 (22.2)	*1*	27 (22)	1 (10)	*0.67*
**Node**						
N +	120 (72.3)	5 (55.6)		87 (69.6)	9 (90)	
N0	46 (27.7)	4 (44.4)	*0.28*	38 (30.4)	1 (10)	*0.28*
**Metastase**						
M0	143 (86.1)	8 (80)		105 (82.7)	10 (100)	
M +	23 (13.9)	2 (20)	*0.64*	22 (17.3)	0 (0)	*0.365*

*Neg* Negative, *Pos* Positive, *n (%)* number and percentage of individuals (except for mean ± SD age), *SD* Standard deviation, *Her-2* Human epidermal growth factor receptor-2, *PR* Progesterone receptor, *ER* Estrogen receptor, *TN* Triple negative

Unknown data were excluded from statistical analysis

^a^bold values are statistically significant (*P* < 0.05). *P* (2 tailed)

^b^both mutations: *p* = 0.002

### Clinical features of BRCA2 c.1310_1313 DelAAGA associated BC

Detailed clinicopathological features of positive patients are listed in Table 2. and the comparisons between carriers and non carriers are summarized in Table 3.

The mean age at diagnosis of BC in patients with *BRCA2* c.1310_1313 DelAAGA mutation was 38.5 years (SD = 8.92, range: 28–54) with a large proportion (60%) diagnosed at 39 years of age or younger compared to noncarriers (37%). All *BRCA2* carriers developed unilateral BC and were more likely to have a family history of BC (70%) when compared to the non carriers (41.3%). Invasive ductal carcinoma is the most common histopathologic type (80%). The occurrence of intermediate tumor lesion T2 (60%), positive lymph nodes (90%), early histological stage (I-II) and no metastasis (0%) was noted in carriers. Only 22.2% (2/12) of the mutation carrier patients are triple-negative.There was no statistically significant difference between positive patients and noncarriers.

### Risk of *BRCA1/2* mutations assessment: Eisinger Score

We aimed at addressing the practice of referral for genetic counseling and establishing whether the Eisinger prediction model could be helpful in identifying individual’s mutation risk and determining eligibility for BC genetic screening in our population.

All patients positive for the two tested mutations (except one case) fit the Eisinger guidelines for BRCA mutation screening (score >  = 3; Table 2). The mean Eisinger Score in positive patients for the founder *BRCA1* c.5309G>T mutation was 7.4 ± 1.18 (median = 7) and 5.63 ± 0.65 (median = 5.5) in patients positive *BRCA2* c.1310_1313 DelAAGA mutation (Table 4). When considering the two mutations together, the mean Eisinger Score in positive patients was 6.61 ± 0.73, (mediane = 6) and that of negative patients ranged from 3.92 ± 0.21 to 4.02 ± 0.20 (mediane = 3) (Table 4).

**Table 4:** Breast cancer BRCA mutations prediction with the Eisinger model

Mutations	Mean score	Median score	Range score	*P* value (M-U_test)
***BRCA1* c.5309G>T**				
Positive (*n* = 10)	7.4 ± 1.18	7	2–16	***0.002***
Negative (*n* = 166)	4.02 ± 0.20	3	0–13	
***BRCA2* c.1310_1313 DelAAGA**				
Positive (*n* = 8) ^a^	5.63 ± 0.65	5.5	3–9	***0.02***
Negative (*n* = 126)	4.02 ± 0.25	3	0–16	
**Both mutations**				
Positive (*n* = 18)	6.61 ± 0.73	6	2–16	***0.00012***
Negative (*n* = 159)	3.92 ± 0.21	3	0–13	

^a^Four patients were excluded because of lack of information

A Mann–Whitney test was then carried out to examine whether or not the Eisinger prediction model was useful in the present population (Table 4). In order to compare the scores effectively, patients who tested positive for the founder mutation (group 1, *n* = 10), or those who tested positive for *BRCA2* deletion mutation (group 2, *n* = 8), as well as the two groups together (group 3, *n* = 18) were compared with patients who tested negative for one or for both mutations. There was a significant difference in the Eisinger risk scores between carriers and non carriers in all three tested groups (*p* = 0.002; *p* = 0.02; *p* = 1.2 × 10–4, respectively).

### Overall survival

**Fig. 1:**
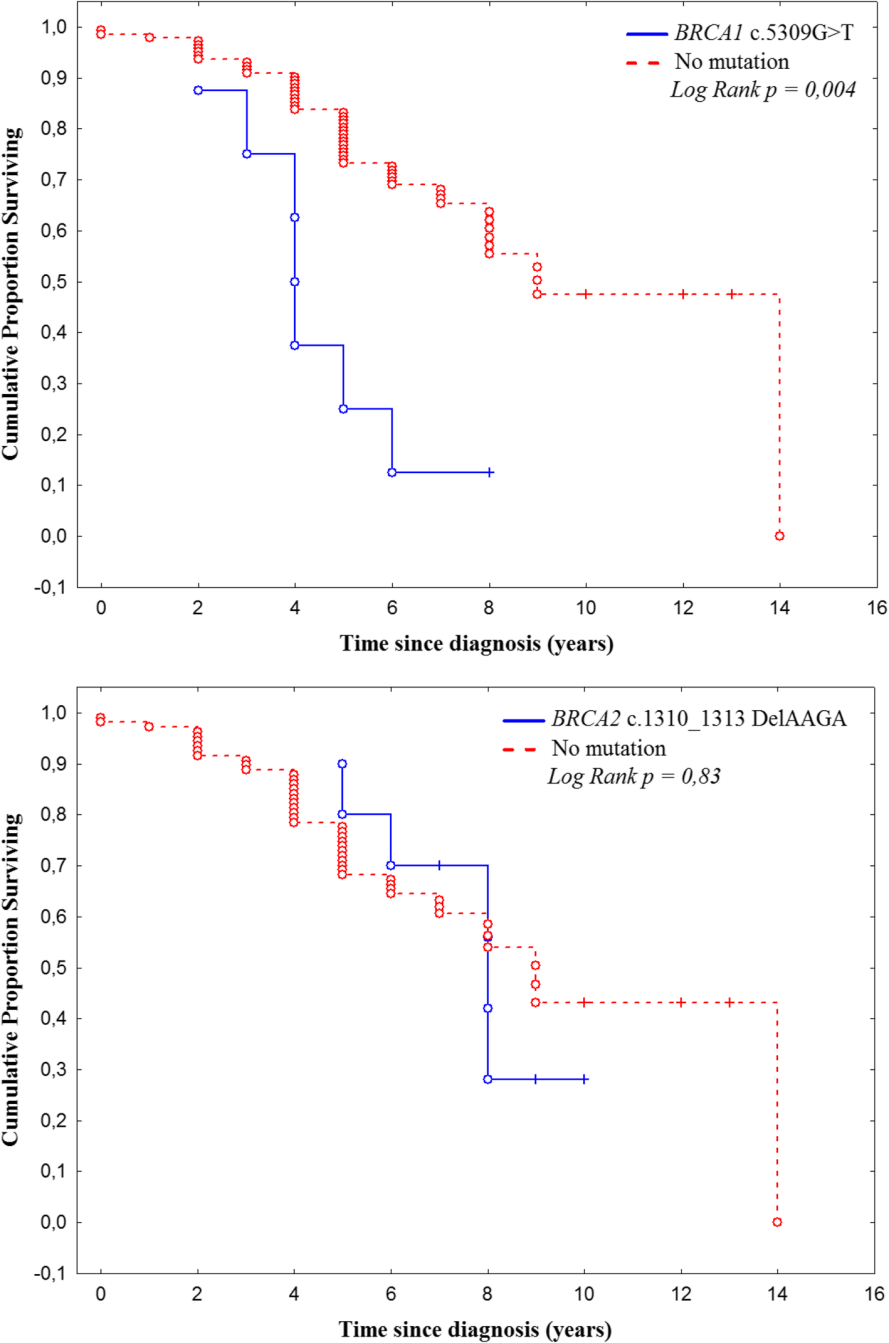
Kaplan–Meier estimates of cummulative survival of patients with *BRCA1* c.5309G>T or BRCA2 c.1310_1313 DelAAGA mutation vs. patients without mutation

In order to determine whether the two BRCA mutations (*BRCA1* c.5309G>T and *BRCA2* c.1310_1313 DelAAGA) affect survival, we compared the overall survival of patients with one BRCA mutation with the non carriers (Fig. 1). Association analysis showed that patients with the *BRCA1* c.5309G > T mutation have worse OS than the negative group (*p* = 0.004). All positive patients are dead except one (data are missing for two patients). On the other side, survival was not significantly affected by the presence of c.1310_1313 DelAAGA mutation (*p* = 0.83).

## Discussion

Genetic testing for pathogenic germline mutations in *BRCA1/2* genes is strongly recommended for people with a BC family history. It may have important implications for clinical management of patients diagnosed with the disease as well as unaffected carriers of these mutations. It also influence prognosis of the current cancer and enable prevention of future cancers [29]. However, genetic testing is expensive and may be associated with adverse psychosocial effects. Identifying most prevalent or founder mutations in specific populations constitute a valuable opportunity for genetic screening since it facilitate earlier and rapid and especially cheaper molecular diagnosis of BC.

Insofar as only a small proportion of people with breast cancer in the general population are carriers of a mutation, it is not possible to propose testing for a mutation at all cases. To provide a cost-efficient and clinically appropriate genetic counseling service, genetic testing should be targeted at those individuals most likely to carry pathogenic mutations. Several algorithms that predict the likelihood of carrying a *BRCA1* or a *BRCA2* mutation are currently used in clinical practice to identify such individuals. Their widespread use would improve equity of access and the cost-effectiveness of genetic testing.

In the current study, we sought to present a first report on the prevalence and clinical significance of two particular *BRCA1/2* mutations in the northeastern region of Morocco. We also evaluated the efficiency of a clinical prediction tool, the Eisinger scoring system, for use in clinical practice to select patients for mutation analysis in our cohort.

The first mutation was *BRCA1* c.5309G>T (p.Gly1770Val; rs863224765) founder mutation that seems to be unique to this region of Morocco. Indeed, this mutation was first reported in two different families from Norway and three in Spain. Interestingly, all of these five families were of Moroccan origin, more precisely from the Moroccan northeast region [22, 30]. To date, only one study reported this mutation in two patients from Tanger (in the north of Morocco) [31]. In addition, the *BRCA1* c.5309G>T mutation was reported as a Moroccan founder variant based on Microsatellite analysis in the five families studied by Quiles et al. [22]. The fact that this variant was never reported in other Moroccan studies nor worldwide, raises the probability of it being specific pathogenic variant for the north eastern Moroccan population.

*BRCA1* c.5309G>T mutation is located in the functionally important *BRCA1* carboxyl terminal (BRCT) domain, a domain known to harbor missense substitutions associated with increased risk of breast/ovarian cancer [32]. Using a functional complementation assay of *BRCA1* sequence variants in a mouse-Brca1-null embryonic stem cells, Bouwman et al. [33] classified this variant deleterious because the gene was functionally impaired in the direct repeat (DR)-GFP and/or combined PARP inhibitor/cisplatin sensitivity assay. Quiles et al. [34] demonstrated that this variant significantly alters the BRCT structure and that it compromises the *BRCA1* transcriptional activity. Further studies based on multifactorial likelihood analysis provided evidence that *BRCA1* c.5309G>T should be treated as a disease-causing variant [32].

The second tested variant in our study was the *BRCA2* c.1310_1313delAAGA frameshift mutation that has been reported as “pathogenic” in the ClinVar (http://www.ncbi.nlm.nih.gov/clinvar/), the Breast Cancer Information Core (BIC) (http://www.research.nhgri.nih.gov/bic/), the HGMD (http://www.hgmd.cf.ac.uk/ac/index.php) databases. This sequence change deletes four nucleotides from exon 10 of the *BRCA2* mRNA, causing a frameshift after codon 437 and the creation of a premature translational stop signal 22 amino acid residues.

*BRCA2* c.1310_1313delAAGA was commonly circulated among north African patients with BC as recurrent mutation since it has been reported in some Moroccan [23, 24], Algerian [35] and Tunisian studies [36, 37]. Interestingly, the genealogic investigation in Moroccan patients revealed that all carriers of this mutation shared the same restricted geographic origin in the North-East of Morocco [24]. Its higher incidence in the north east of Morocco is suggestive of a founder effect which required confirmation by haplotype analysis. Moreover, this mutation was also found in other patients from European [38–42], Hispanic [43], Libanese [44] and Caribbean [45] origins.

In our cohort, all Moroccan BC patients were originating from the northeastern region of Morocco and were grouped into familial (47%) and sporadic (53%) groups using Eisinger scoring system. All of the patients were screened for the two mutations *BRCA1* c.5309G>T and *BRCA2* c.1310_1313delAAGA and the clinicopathological features of carriers were analyzed.

The first main finding was the high prevalence of these two mutations in our cohort (12.5%). The screening of *BRCA1/2* genes by NGS sequencing confirmed that there are no additional mutations detected among positive patients for *BRCA1* c.5309G>T or *BRCA2* c.1310_1313delAAGA. The two mutations appeared to be the most frequent genetic cause of BC in our population preferentially in a strong familial context of the disease since it can explain at least 20% of familial cases. This result concur, as it has been documented by others, that the family history is an important criterion for the identification of *BRCA1/2* mutation carriers [46, 47].

In addition, the cohort we screened for *BRCA2* c.1310_1313delAAGA mutation included 4 BC males and among them, two are positive for this alteration. Interestingly, this mutation has also been previously detected in one Tunisian male [36]. This may suggest that this mutation has a relatively high penetrance in males. Male breast cancer (MBC) is a rare disease accounting for less than 1% of all breast cancer cases and it was previously shown that nearly 90% of MBC arising in BRCA mutation carriers are found to harbor a *BRCA2* mutation [48].

Unexpectedly and interestingly, we found (work in progress in an ongoing study in our laboratory) that the screening of *BRCA1/2* genes using NGS method, starting with 54 familial BC patients, revealed a reduced mutational landscape, characterized by the presence of 4 different mutations detected in 47% of the patients tested. Here, *BRCA1* c.5309G>T and *BRCA2* c.1310_1313delAAGA mutations accounted for the majority (92%) of these mutations. Taken together, these observations suggest either that a small number of mutations exist in this population, although increasing the number of patients is necessary to prove this hypothesis (this work is in progress using NGS to screen a larger number of patients), or that the diversity of the mutations in this population is far from being known.

The occurrence of several breast cancers in the same family is an example of a family history that may signal a genetic predisposition. When a breast cancer risk is suspected, it should lead to genetic counseling. The Moroccan population has limited access to clinical genetics and information regarding genetic counseling is usually delivered by other health-care professionals like oncologists.

The Eisinger’s score assessment [26] is a family tree analysis and is used here to decide whether an oncogenetic consultation is advisable. This scoring model has the advantage of being simpler and allowing the clinician to quickly identify patients for referral to oncogenetic counseling [49].

We have seen in our study that a threshold of 3 for the Eisinger score made it possible to diagnose 94.44% of patients with the disease carrying one of the two *BRCA1/2* mutations tested here. Low score observed in one case was probably reflecting a lack of information rather than an absence of cancer occurring within these families. Nevertheless, these findings provide substantial evidence that the Eisinger scoring system is an efficient screening tool method to identify counselees BC patients at high risk for hereditary breast cancer for genetic counseling. Incorporating this carrier prediction algorithm into risk assessment may improve breast cancer management in our population.

On another side, we evaluated the phenotypic spectrum of BC according to the presence of *BRCA1* c.5309G>T and *BRCA2* c.1310_1313delAAGA mutations. It is noticeable that patients carrying these mutations are more prone to have early breast cancer onset. Although some patients were diagnosed at a relatively middle age (6 patients with age > 45 years), they have family members with BC diagnosed at younger age. Thereby, the relatively middle age of indexes could be due to delay in access to care services rather than late onset of disease.

The clinical features of most *BRCA1* c.5309G>T related tumors were more frequently of the ductal invasive type, with intermediate grade and half of them were triple negative. This is in accordance with typical characteristics of *BRCA1* pathogenic mutations [18, 22].

Although there was not statistically differences between *BRCA1* c.5309G>T carriers group and *BRCA2* c.1310_1313delAAGA carriers group in terms of tumor laterality, histopathological subtype, tumor grade, RE and RP expression, we detected proportional differences between patients groups. It was found that *BRCA1* c.5309G>T mutation showed more aggressive features than *BRCA2* c.1310_1313delAAGA in terms of triple negative status and overall survival. Indeed, we found that patients with *BRCA1* c.5309G>T mutation had a significantly worse prognosis regarding OS than negative group. All positive patients are dead except one. On the other hand, *BRCA2* c.1310_1313delAAGA mutation carriers were more likely to be diagnosed with breast cancer already spread to regional lymph nodes. Although the overall survival was not statistically affected by the presence of this deletion, there was a trend to reduce survival in many cases (death was reported in 7/12 patients (58%)).

These data indicated that BC patients with *BRCA1* c.5309G>T and *BRCA2* c.1310_1313delAAGA mutations had poor survival outcomes and hence screening patients with BC for BRCA mutations might help in strategizing their treatment and improving their survival. For persons carrying a constitutional genetic impairment related to HBOC syndrome, an appropriate follow-up strategy based on surveillance and/or preventive surgery should be provided. Performing prophylactic mastectomy to reduce the risk of contralateral breast cancer and prophylactic ovariectomy are two types of surgery proposed to women with *BRCA1* and BCRA2 gene abnormalities [50]. Quiles et al. [22] who first described *BRCA1* c.5309G>T mutation adopted the following protocol: In general, patients are offered prophylactic mastectomy and prophylactic salpingo-oophorectomy. Annual breast magnetic resonance imaging and mammography is an alternative if the patients do not wish to undergo prophylactic mastectomy. Breast surveillance is offered from the age of 25 and oophorectomy from the age of 35.

## Conclusion

The knowledge about the contribution of *BRCA1* and *BRCA2* mutations in Moroccan BC will lead to better understanding of genetic risk factors of this disease. Altogether, the data in this study indicated that the *BRCA1* c.5309G>T and *BRCA2* c.1310_1313delAAGA mutations are currently the most significant BRCA genetic cause of BC in Northeastern Moroccan population. Systematic screening for this mutation in BC patients should be considered to facilitate early detection of subjects at high risk of BC among their relatives.

Our study indicates that the Eisinger scoring model could be recommended to decide whether an oncogenetic consultation is advisable to assess the probability of a *BRCA1* or *BRCA2* mutation.

To our best knowledge, this work represents the first study in North east of Morocco supporting the major contribution of the *BRCA1* c.5309G>T and *BRCA2* c.1310_1313delAAGA mutations to BC. However, we believe that the sample size is still small and larger cohorts are needed to trace a clear and complete *BRCA1/2* mutational spectrum in this population.

## Data Availability

The datasets produced and analysed in the present study were deposited in the NCBI Sequence Read Archive. They can be accessed publicly using the BioProject accession number PRJNA949945 and the BioSample accession numbers SAMN33963498 to SAMN33963539. Additionally, the raw Sanger reads of all samples are available for retrieval on the Harvard Dataverse website through the following links: https://doi.org/10.7910/DVN/B5DG3Q and https://doi.org/10.7910/DVN/DAESLS.

## Abbreviations

BC: Breast cancer
*BRCA1/2*: Breast Cancer genes 1/2
ER: Estrogen receptor
Her-2: Human epidermal growth factor receptor 2
NGS: Next generation Sequencing
OS: Overall surviving
PR: Progesterone receptor
